# The complete mitochondrial genome of the firefly, *Diaphanes citrinus* (Olivier), (coleoptera: Lampyridae)

**DOI:** 10.1080/23802359.2019.1664351

**Published:** 2019-09-16

**Authors:** Zhuo Yang, Xinhua Fu

**Affiliations:** a Hubei Insect Resources Utilization and Sustainable Pest Management Key Laboratory, College of Plant Science and Technology, Huazhong Agricultural University, Wuhan, China;; b Firefly Conservation Research Centre, Wuhan, China

**Keywords:** *Diaphanes citrinus*, firefly, lampyridae, mitochondrial genome

## Abstract

We report the complete mitochondrial genome of firefly, *Diaphanes citrinus* (Olivier). The circular genome of 18,594 bp and has a base composition of A (41.26%), C (11.64%), G (10.18%), T (36.92%). Similar to other Metazoa, our sequence contains 13 protein-coding genes, 21 transfer RNA genes, two ribosomal RNA genes, and a non-coding AT-rich region. We sequenced the mitochondrial genome of fireflies to analyze phylogenetic relationships and deduce the evolution of their flashing signals.

## Introduction

Firefly flashing has fascinated humankind for millennia, and its mystery has been gradually solved (Trimmer et al. 2001; Ghiradella and Schmidt 2004; Aprille et al. 2004). The genus *Diaphanes,* was established by Motschulsky (1853), has over 90 species recorded from the Oriental and the Ethiopian realms (Olivier 1907, 1910; Mcdermott 1964, 1966). Among this spieces, *Diaphanes citrinus* is uniqe by its diaphanous area, alongate body, and its special orange coloration (Jeng et al. 2001; Fu 2014).

Mitochondrial genome sequences are essential to a deeper understanding of the evolution of Lampyridae and other luminescent beetles (Ermakov et al. 2006). Here, we elucidate the mtDNA genome of *D. citrinus.*

Specimens used in this study were collected from Huanjiangmulun attractions, Hechi City, Guangxi Province(25°05′N, 107°93′E), and were stored in Natural History Museum, Huazhong Agricultural University, Wuhan, Hubei, China (its accession no. DC2014092405).

Specific primers were designed based on these conserved regions sequences. The PCR reaction was carried out with LA Taq polymerase for 35 cycles at 94 °C for 30 s, and annealedat 50 °C for 30 s, followed by extension at 72 °C for 1 min per 1 kb. Sequences were assembled using the software DNAstar v7.1 (DNAstar, Madison, WI) and adjusted manually to generate the complete sequence of mitochondrial DNA.

The complete mitochondrial genome sequence of *D.citrinus* (GenBank:MH651351) has 18594 bp and has a base composition of A (41.26%), C (11.64%), G (10.18%), T (36.92%). Similar to other Metazoa, our sequence contains 13 protein-coding genes, 21 transfer RNA genes, two ribosomal RNA genes, a non-coding AT-rich region and one tandem repeats unit(TRU), which represents a typical insect mitochondrial genome (Wolstenholme 1992). The open frames of the 13 protein-coding genes were inferred from three other fireflies:*Aquatica leii*, *Luciola substriata*, and *Pyrocoelia rufa* (Lee et al. 2003; Jiao et al. 2015; Mu et al. 2016). All 13 PCGs initiated with ATN (ATT, ATA, and ATG) codon. Among those genes, four PCGs (ATP6, ND4, ND4L, CTYB) initiated from ATG, and five PCGs (COI, ND2, ND5, ND6, ND1) initiated from ATT, and four PCGs (COII, COIII, ND3, ATP8) initiated from ATA. In addition, an incomplete terminal codon namely single T was found in five PGGS (COII, COIII, ND5, ND4, ND6), In case of other eight PGGS, TAA (ND2, COI, ATP8, ATP6, ND4L) or TAG (ND3, CTYB, ND1) was used.

**Figure 1.**
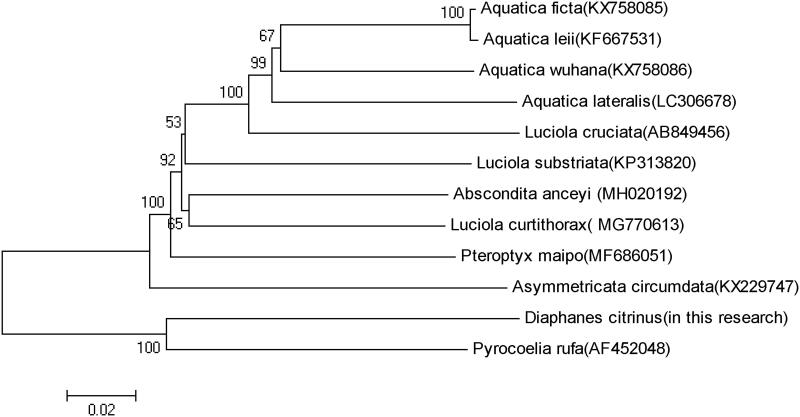
Molecular phylogeny of *D.citrinus* and 11 other firefly species based on the complete mitochondrial genome. The complete mitochondrial genome was downloaded from GenBank and the phylogenic tree was constructed by neighbour-joining method with 1000 bootstrap replicates. MtDNA accession numbers used for tree construction are as follows: *Pteroptyx maipo* (MF686051), *Aquatica ficta* (KX758085), *Pyrocoelia rufa* (AF452048), *Aquatica leii* (KF667531), *Luciola curtithorax* (MG770613), *Aquatica wuhana* (KX758086),* Luciola cruciata* (AB849456), *Asymmetricata circumdata* (KX229747), *Aquatica lateralis* (LC306678), *Abscondita anceyi* (MH020192) and *Luciola substriata* (recently identified as *Sclerotia flavida* by Ballantyne et al. 2016)(KP313820).

The phylogenetic tree among the 12 species based on mitochondrial genome sequences were aligned in MEGA 5(Phoenix, AZ) (with 1000 bootstrap replicates) to construct a Neighbour-Joining tree (Figure 1). The result shows *D.citrinus* is most closely related to *Pyrocoelia rufa,* which belongs to an entirely different genus in the Lampyridae.

In conclusion, the complete mitochondrial genome sequence of *D.citrinus* provides an important molecular framework for further phylogenetic analyses of fireflies.

## References

[CIT0001] Aprille JR, Lagace CJ, Modica-Napolitano J, Trimmer BA 2004 Role of nitric oxide and mitochondria in control of firefly flash. Integr Comp Biol. 44:213–219. 21676698 10.1093/icb/44.3.213

[CIT0002] Ballantyne LA, Lambkin CL, Luan XIN, Boontop Y, Nak-Eiam S, Pimpasalee S, Silalom S, Thancharoen A 2016 Further studies on south eastern asian luciolinae: 1. Sclerotia Ballantyne, a new genus of fireflies with back swimming larvae 2. Triangulara Pimpasalee, a new genus from Thailand (Coleoptera: Lampyridae). Zootaxa. 4170:201. 27701260 10.11646/zootaxa.4170.2.1

[CIT0003] Ermakov OA, Surin VL, Titov SV, Zborovskiĭ SS, Formozov NA 2006 A search for Y-chromosomal species-specific markers and their use for hybridization analysis in ground squirrels. Genetika. 42:538–548. 16756073

[CIT0004] Fu XH 2014 An illustrated handbook of Chinese fireflies. Beijing (China): The Commercial Press p. 167 (in Chinese).

[CIT0005] Ghiradella H, Schmidt JT 2004 Fireflies at one hundred plus: a new look at flash control. Integr Comp Biol. 44:203. 21676697 10.1093/icb/44.3.203

[CIT0006] Jeng ML, Lai J, Yang PS, Sat M 2001 Revision of the genus *Diaphanes motschulsky* (Coleoptera: Lampyridae: Lampyrinae) of Taiwan. Jpn J Syst Entomol. 7:203–235.

[CIT0007] Jiao H, Ding M, Zhao H 2015 Sequence and organization of complete mitochondrial genome of the firefly, *Aquatica leii* (Coleoptera: Lampyridae). Mitochondrial DNA. 26:775–776. 24289147 10.3109/19401736.2013.855746

[CIT0008] Lee S-C, Bae J-S, Kim I, Suzuki H, Kim S-R, Kim J-G, Kim K-Y, Yang W-J, Lee S-M, Sohn H-D, Jin B-R 2003 Mitochondrial DNA sequence-based population genetic structure of the firefly, *Pyrocoelia rufa* (Coleoptera: Lampyridae). Biochem Genet. 41:427–452. 14994830 10.1023/b:bigi.0000007777.87407.1b

[CIT0009] McDermott FA 1964 The taxonomy of the Lampyridae (Coleoptera). Trans Am Entomol Soc. 90:1–72.

[CIT0010] McDermott FA 1966. Lampyridae, In STEEL. W. O.(ed. ) Coleopterorum Catalogus Supplementa, prs 9(ed. secunda). iii+149 pp. W. JUNK,'s-Gravenhage.

[CIT0011] Mu FJ, Ao L, Zhao HB, Wang K 2016 Characterization of the complete mitochondrial genome of the firefly, *Luciola substriata* (Coleoptera: Lampyridae). Mitochondrial DNA A DNA Mapp Seq Anal. 27:3360–3362. 25714154 10.3109/19401736.2015.1018221

[CIT0012] Olivier E 1907 Coleoptera. Fam. Lampyridae In: Wytsman P, editor. Genera insecttorum, Fasc. 53 Brussels: Verteneull and desmet; p. 74.

[CIT0013] Olivier E 1910 Description de lampyrides nouveaux (Col.). Bull Soc Ent France. 15:285–286.

[CIT0014] Trimmer BA, Aprille JR, Dudzinski DM, Lagace CJ, Lewis SM, Michel T 2001 Nitric oxide and the control of firefly flashing. Science. 292:2486. 11431567 10.1126/science.1059833

[CIT0015] Wolstenholme DR 1992 Animal mitochondrial DNA: structure and evolution. Int Rev Cytol. 141:173–216. 1452431 10.1016/s0074-7696(08)62066-5

